# Microstructure, Mechanical Properties, and Corrosion Behavior of Ultra-Low Carbon Bainite Steel with Different Niobium Content

**DOI:** 10.3390/ma14020311

**Published:** 2021-01-09

**Authors:** Yun Zong, Chun-Ming Liu

**Affiliations:** 1 Key Laboratory for Anisotropy and Texture of Materials, Ministry of Education, Northeastern University, Shenyang 110819, China; 2 School of Mechanical and Automobile Engineering, Qilu University of Technology (Shandong Academy of Sciences), Jinan 250353, China; 3 School of Materials Science and Engineering, Northeastern University, Shenyang 110819, China

**Keywords:** ultra-low carbon bainite steel, niobium microalloying, mechanical property, corrosion resistance, corrosion behavior

## Abstract

Four types of ultra-low carbon bainite (ULCB) steels were obtained using unified production methods to investigate solely the effect of niobium content on the performance of ULCB steels. Tensile testing, low-temperature impact toughness testing, corrosion weight-loss method, polarization curves, electrochemical impedance spectroscopy (EIS), and the corresponding organizational observations were realized. The results indicate that the microstructure of the four steels comprise granular bainite and quite a few martensite/austenite (M/A) elements. The niobium content affects bainite morphology and the size, quantity, and distribution of M/A elements. The elongation, yield strength, and tensile strength of the four types of ULCB steels are above 20%, 500 MPa, and 650 MPa, respectively. The impact toughness of the four types of ULCB steels at −40 °C is lower than 10 J. Steel with Nb content of 0.0692% has better comprehensive property, and maximum charge transfer resistance in 3.5 wt.% NaCl solution at the initial corrosion stage. The corrosion products on the surface of steel with higher niobium content are much smoother and denser than those steel with lower niobium content after 240 h of corrosion. The degree of corrosion decreases gradually with the increase of niobium content at the later stage of corrosion.

## 1. Introduction

Ultra-low carbon bainite (ULCB) steel is a kind of multipurpose steel that has attracted wide attention owing to its outstanding comprehensive properties such as high strength and toughness, better weldability, and other characteristics [1,2,3,4,5,6,7,8]. The performance of the steel is directly relevant to the composition, production process, and corresponding microstructure [9]. The composition of ULCB steel does not require more carbon and alloying elements to maintain strength. Its superior performance can be obtained by a low-carbon microalloying composition and advanced production methods such as controlled rolling [10,11]. The strength of steel mainly depends on dislocation strengthening in bainite structure, fine crystal strengthening, precipitation strengthening and micro-alloy strengthening. The design of lower carbon content and the lower total amount of alloys results in an excellent comprehensive performance and overcomes the contradiction between material performance, cost, profit, and energy consumption. Currently, low-carbon bainitic steel is the choice of steel for offshore platforms [12].

When ULCB steel is used in the construction of offshore platforms under marine environment or at low temperatures, its superior mechanical and welding properties as well as certain seawater corrosion resistance are necessary. Some research has indicated that the strength, hardness, and plasticity of steel can be significantly improved, and the corrosion rate can be remarkably reduced by the addition of micro-alloyed elements [13,14]. Generally, appropriate amounts of copper, nickel, and chromium are added to ULCB steel for effectively improving the corrosion resistance [15,16,17,18,19]]. Niobium, an important micro-alloyed element, is also added for strength and improvement of the low-temperature toughness in steel for both marine engineering structures and liquefied petroleum gas (LPG) transport ship hulls [20]. Niobium is considered particularly critical because of its multiple effects on ULCB steel. Trace niobium can increase the strength, toughness, and ductility of steel by multiple mechanisms [21,22,23,24]. However, it has been reported that the effect of niobium content on steel weldability is on both sides [20,21]. The addition of niobium must be moderate in range. Moreover, the change in niobium content causes a change in the chemical composition, which significantly affects the phase transition behavior and the corresponding microstructure. The composition and microstructure play an important role for determining the corrosion susceptibility of steel [25,26,27]. However, the effect of niobium content on the seawater corrosion resistance of ULCB steel is still unclear. Because there are complex interactions between chemical composition, deformation, the phases formed, and microstructural unit sizes and homogeneity, the properties of steel are affected by several factors. Thus far, there have been relatively few studies on the influence of Nb content on the microstructure and properties of ULCB steel used in offshore platforms, excluding other factors. In addition, the correlation between niobium content and the extent to which it affects performance, particularly corrosion resistance, is ambiguous; furthermore, the niobium content appropriate for a favorable comprehensive performance in offshore platform steel remains unclear. The cost-effective design and production of value-added niobium-bearing offshore platform steel with an excellent comprehensive performance is in progress.

To investigate the microstructure, mechanical properties, seawater corrosion resistance, and corrosion behavior of ULCB steel with various niobium contents and to reveal the correlation between them, the design of low-carbon micro-alloyed composition with different Nb contents is adopted in this study. All steel samples were obtained by unified production and the preparation methods were intended to investigate the effect of niobium content only on the properties, excluding other factors. Then, the strength, plasticity, and impact toughness at −40 °C, as well as corrosion resistance in three and a half percent mass fraction (3.5 wt.%) NaCl solution were examined. In addition, the corresponding microstructure and surface morphology were observed.

This study provides a beneficial reference for the design and manufacture of low-carbon bainite steel and its application in full immersion marine environments.

## 2. Materials and Methods

### 2.1. Materials

Based on the low carbon content, trace niobium, titanium, copper, nickel, chromium, and molybdenum were added to ensure the acquisition of bainite structure and excellent comprehensive performance. To investigate the effect of Nb content on the microstructure and properties of low-carbon bainitic steel, the content of Nb in four types of steels was 0.035%, 0.062%, 0.084%, and 0.107%, respectively. Other elements were present in same quantities in all four samples. The chemical compositions of the four types of experimental steels with different niobium contents are listed in Table 1.

The experimental steel was smelted in a 50 kg vacuum furnace (Made in Liaoning Jinzhou North Electric Furnace Equipment Co. Ltd, Jinzhou, China) and rolled on a 300 mill. The size of ingots was 80 mm × 80 mm ×200 mm, and the thickness of rolled plates was 14 mm. Multiple passes of high temperature cumulative deformation at 1050–950 °C were adopted, and the total reduction ratio was 82.5%. After rolling, the plates were cooled at 850–550 °C with a fast-cooling process, and then cooled to room temperature (25 ± 1 °C) in air.

### 2.2. Methods

**Figure 1:**
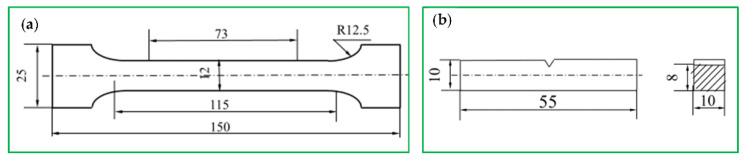
Size of samples for (**a**) tensile test and (**b**) impact test (mm).

Samples were taken from the four rolled steel plates. Tension samples and impact samples were machined into the size, as shown in Figure 1.

Uniaxial tension tests were performed with a CMT5105 SANS microcomputer control electronic universal testing machine (New Sans (Shanghai) Enterprise Development Co., Ltd., Shanghai, China). The impact toughness at −40 °C was tested based on the standard of the American Society for Testing Material (ASTM) E2298 [28]. The Vickers hardness (HV) was measured using HVS-50 (Laizhou Huayin Experimental Instrument Co., Ltd., Laizhou, China) under 98 N load with 15 s dwell time according to ASTM E384 [29]. Three parallel samples were utilized to test the same observation point.

The sample size for the polarization curve and impedance testing was machined into 10 mm × 10 mm × 2 mm specimen. The sample size for the corrosion weight-loss experiment was machined into 25 mm × 10 mm × 2 mm. All samples were ground up to 1500 grit, washed with deionized water and alcohol by ultrasonic sound, degreased with acetone, and dried in a desiccator.

The electrochemical tests were conducted using a three electrode system. The experimental steel sample, platinum-coated niobium mesh electrode, and saturated calomel electrode (SCE) were the working, auxiliary, and reference electrodes, respectively. A copper wire was welded on the back of each polarization curve and impedance test sample as an electrical connection, exposing a square (10 mm × 10 mm) surface on the front. The rest of the sample surface was sealed with paraffin. Samples were completely immersed and suspended in 3.5 wt.% NaCl solution at room temperature (25 ± 1 °C). The electrochemical tests were conducted using an electrochemical workstation (Interface 1000, Gamry Instruments, Inc., Warminster, PA, USA). The electrochemical impedance spectroscopy (EIS) results were obtained by employing an AC voltage with an amplitude of 10 mV in the frequency range 0.01–100,000 Hz. The test parameters of the polarization curve ranged from −1 mV to +1 mV, and the scanning rate was 0.5 mV·s^−1^.

The corrosion weight-loss method was used to determine the corrosion rates and study the kinetics of corrosion. Corrosion samples of all four types of steels underwent 24 h, 72 h, 168 h, 240 h, and 360 h of corrosion, respectively. The corrosion samples were grouped according to the experimental scheme. The corrosion samples were grouped according to the experimental scheme. Four samples were taken from each observation point, three of which were used for the weight loss test, and the other one was used for scanning electron microscopy to observe the surface morphology of rust layer. The samples were weighed before soaking. Then, the sample was suspended vertically in a solution with an insulating plastic cord. The samples were separated from each other at a certain distance, each sample was kept on almost the same level and immersion time was recorded. During corrosion, the macroscopic morphological changes of the sample surface were observed. After the samples were taken out, the loose corrosion products on the sample surface were brushed with a bristle brush. Then, three parallel samples were selected from each group and polished with 800# sandpaper. The remaining corrosion products on the surface were removed without damaging the sample matrix. After the corrosion products were removed, ultrasonic cleaning with deionized water was performed for five minutes to remove surface impurities. They were then cleaned and dehydrated with anhydrous ethanol. Finally, the samples were blown dry and weighed.

The corrosion rate can be calculated as follows [30,31]:

(1) $$v^{-}=\left(m_{0}-m_{1}\right)/S\times t$$

where $v^{-}$ is the corrosion rate of the experimental steel (g·m^−2^·h^−1^); *m*_0_ denotes the weight of the sample before corrosion (g); *m_1_* refers to the weight of the sample after rust removal (g); *t* is the corrosion time (h); and *S* is the area of sample surface in corrosion test (m^2^).

The metallographic structures and surface morphology of the corrosion products on the experimental steel were made by optical microscopy LEICA DM2700M (Leica Microsystems Inc., Wetzlar, GER) and SEM QUANTA 200 (FEI Company of the Netherlands, Eindhoven, The Netherlands).

## 3. Results

### 3.1. Microstructure of Four Types of Experimental Steels

**Figure 2:**
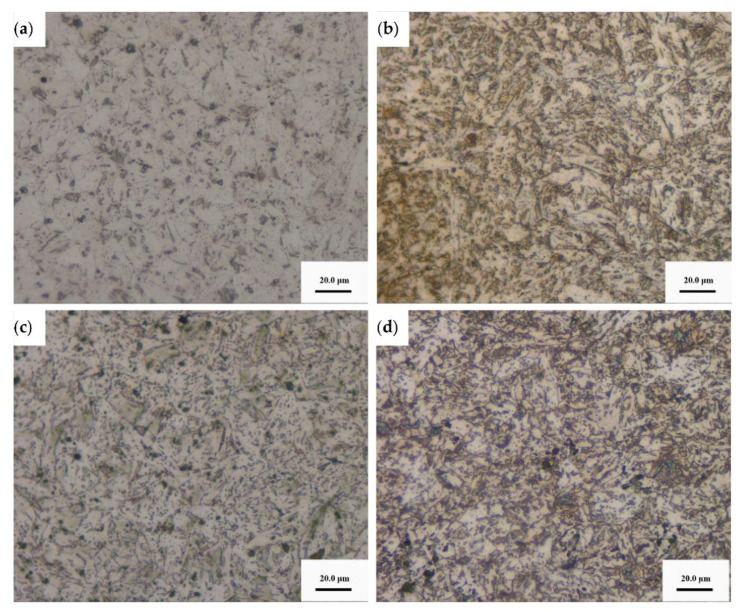
Metallographic structures of the four types of niobium bearing steels (**a**) No. 8 steel (**b**) No. 9 steel (**c**) No. 10 steel (**d**) No. 11 steel.

Metallographic structures of the four types of niobium bearing steels are shown in Figure 2. The microstructures of the four kinds of ULCB steel mainly consist of granular bainite, and a certain number of martensite/austenite (M/A) components are distributed on the matrix. With increase in niobium content, the grain shape of bainite changes from a multilateral shape to an angular shape, and the size reduces. The content, size, and distribution of M/A elements are different for the four types of steels. In steel with lower niobium content, the M/A components are less in number and larger in size. However, the M/A components in steel with higher Nb content, are more abundant and smaller in size.

The content, size, and distribution of the M/A components depend on the composition of steel, cooling temperature, cooling strength, and other deformation processes. A large amount of M/A structures in low-alloy steel destroys the integrity of steel, affects the properties of steel, and is detrimental to the toughness and plasticity of steel. In addition, the M/A components also affect the corrosion resistance of bare steel.

### 3.2. Mechanical Properties of Four Types of Experimental Steels

**Figure 3:**
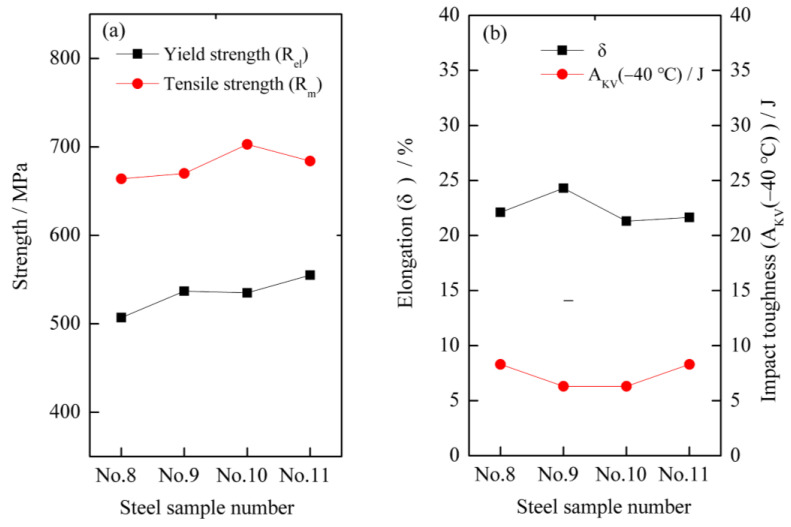
Mechanical properties of four types of ULCB steels (**a**) Yield strength (R_el_) and Tensile Scheme (R_m_) (**b**) Elongation (δ) and −40 °C impact toughness (A_KV_ (−40 °C)).

The mechanical properties of the four types of ULCB steels are shown in Figure 3. The four types of ULCB steels in as-rolled state have higher strength and better plasticity but lower impact toughness at −40 °C. The elongation (δ), yield strength (Rel), and tensile strength (R_m_) of the three performance indexes are above 20%, 500 MPa, and 650 MPa, respectively. However, the Charpy V-Notch (CVN) impact toughness at −40 °C (A_KV_ (−40 °C)) of the four types of experimental steels in the as-rolled state did not vary significantly with the change in niobium content and is lower than 10 J. The yield strength of No. 8 steel with an average of 507 MPa is the lowest. The yield strength of No. 11 steel is the highest, reaching 555 MPa. The yield strength of No. 10 steel is similar to that of No. 9, which is approximately 535–537 MPa. This suggests that the Rel of all experimental steels increases with niobium content. No. 10 steel has the highest tensile strength, exceeding 700 MPa. The tensile strength of steel No. 8, 9, and 10 increases in turns. However, the tensile strength of steel No. 11 with a niobium content of 0.107% is lower than that of No. 10 with a niobium content of 0.084%.

The elongation of steel No. 8 and 9 with lower niobium content is higher than that of No. 10 and 11 with higher niobium content. The elongation of steel No. 9 exceeds 24%, which is the largest among the four types of experimental steels.

Evidently, the niobium content has a vital influence on the strength and plasticity of steel; however, the effect of niobium content on the strength and plasticity is not monotonic, but complex. Therefore, an optimal value of niobium content exists for both strength and plasticity. Among the four experimental steels, No. 9 with 0.0692% niobium content has a better comprehensive property of strength and elongation.

The low-temperature toughness of the low-carbon bainite steel in the as-rolled condition is not ideal in a low-temperature resistance environment. To obtain a perfect comprehensive performance, appropriate heat treatment must be adopted, particularly for steel plates with large thicknesses. The reason is that if only thermal mechanical rolling is used to produce this type of steel, it is liable to be limited by the capacity of the rolling mill equipment and the cooling capacity of the cooling equipment. Under these circumstances, it is difficult to ensure sufficient pass pressure and cooling rate. Consequently, it can easily cause uneven microstructure in the thickness and damage the strength, lamellar tearing resistance, and low temperature toughness. Therefore, the application of reasonable heat treatment is indispensable to produce large-thickness ULCB steel plates.

### 3.3. The Initial Corrosion Behavior and Corrosion Resistance of Four Experimental Steels in 3.5 wt.% NaCl Aqueous Solution

**Figure 4:**
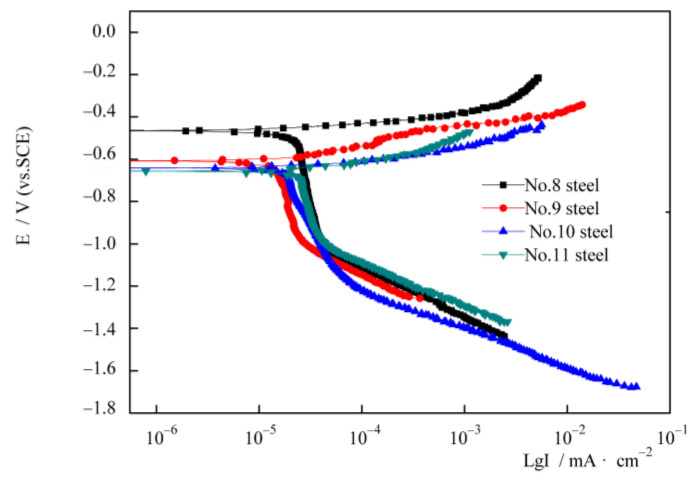
Polarization curves of four types of experimental steels exposed to 3.5 wt.% NaCl aqueous solution.

The polarization curves of the four types of experimental steels exposed to 3.5 wt.% NaCl aqueous solution in the initial soaking stage are shown in Figure 4. The polarization curves of the four types of steels have similar characteristics. With the increase in current density, the anode precipitation potential increases and the cathode precipitation potential decreases.

The polarization curve was fitted using Tafel’s equation [31]. The electrochemical parameters obtained by fitting are listed in Table 2. It is revealed that the corrosion potential (E_0_) becomes more negative with the increase in Nb content. The values of corrosion current density (I_0_) of the four types of steels are of the same magnitude order, and the difference is not evident. The I_0_ values of steel No. 8 and 11 are slightly smaller than those of steel No. 9 and 10. Steel No. 9 with 0.0692% Nb has the smallest corrosion current density compared to other experimental steels in the initial corrosion stage.

The cathodic part of the polarization curve is due to the limiting current density determined by the oxygen reduction reaction. Oxygen plays an important role in cathodic corrosion [30].

There are two evident inflection points on the cathode section of the polarization curve. The cathode oxygen reduction is divided into three stages. In the first stage, the potential of the metal is positive, and the cathode reaction is controlled by the activation polarization of oxygen. In the second stage, the metal potential is negative, and the cathode reaction changes from the oxygen activation polarization control to oxygen diffusion control. The rate of corrosion is limited by the rate of oxygen diffusion. In the third stage, the potential of the metal is more negative, and the cathode reaction of the metal material changes from the oxygen concentration diffusion control to hydrogen evolution activation control.

In the initial stage, the corrosion rate of bare steel is higher. This is because there is abundant dissolved oxygen in the corrosion solution, and the bare steel surface is in direct contact with the corrosion solution. At this stage, the electrochemical reaction is active, so the corrosion rate is higher. Then, the electrochemical reaction proceeds continuously. The dissolved oxygen is constantly consumed and reduces gradually. Thus, the cathode reaction is inhibited, and the corrosion rate falls rapidly. After that, more corrosion products are produced on the bare steel surface that prevent the sample surface from contacting the corrosion solution, which further inhibits the corrosion [19]. Therefore, the corrosion rate decreases gradually.

### 3.4. Electrochemical Impedance Spectroscopy of Four Types of ULCB Steels in 3.5 wt.% NaCl Solution

**Figure 5:**
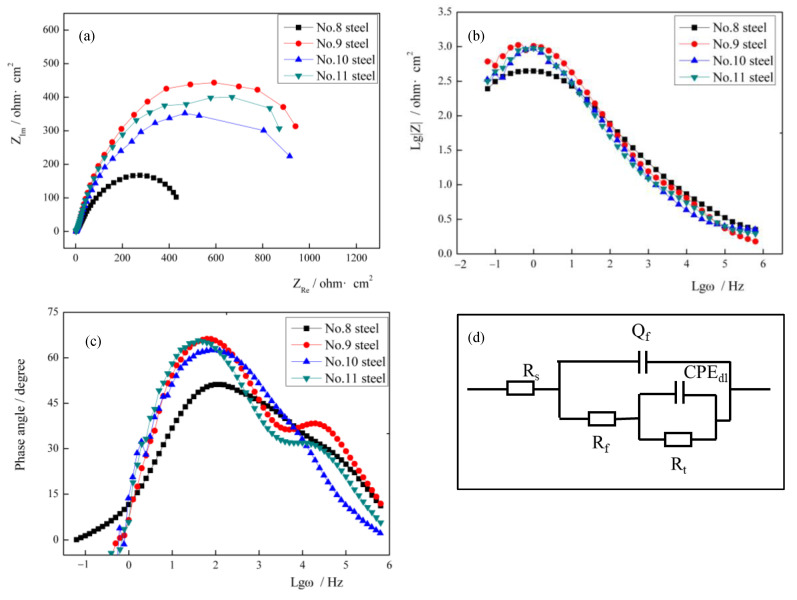
(**a**) Nyquist plots, (**b**) Lg Z—Lg ω Bode plots and (**c**) phase angle—Lg ω Bode plots of four types ULCB steels samples with different content of niobium in 3.5 wt.% NaCl solution and (**d**) equivalent circuit to fitting EIS data (Here, Rs represents solution resistance; Cc and Rc refer to the capacitance of corrosion products film and its resistance, respectively; Cd is the capacitance of double electrode layer, and Rt stands for charge transfer resistance).

EIS was applied to explore the corrosion kinetics of the four types of ULCB steels. The Nyquist and Bode plots of the four types of ULCB steels are presented in Figure 5. As shown in the Nyquist plots, the impedance spectra of the four types of ULCB steels have similar shapes, demonstrating that they have the same corrosion mechanisms and processes in the initial stage. All the Nyquist plots consist of a clear capacitive reactance arc. This indicates that activation controls the corrosion reaction. The high-frequency part in the capacitive reactance arc reflects the impedance information between the corrosion solution and corrosion products generated on the steel surface, which corresponds to the double layer behavior and the change in solution resistance (R_s_) at the original reaction interface of the four types of steel substrates. The low-frequency part in the capacitive reactance arc shows the impedance information of the steel electrode surface; hence, the low-frequency part reflects the corrosion behavior of the electrode surface.

Among the four types of ULCB steels, the steel with the smallest capacitive arc reactance radius is the steel No. 8, which has the lowest niobium content, and the steel with the largest capacitive arc reactance radius is the steel No. 9. Generally, a large capacitive arc diameter represents a large charge transfer resistance (R_t_). R_t_ describes the interfacial resistance between the steel and the corrosion product layer, which is the final barrier that prevents chlorine ions from reaching the metal surface [32]. A large capacitive arc diameter means that corrosion products have a strong protective ability, and the degree of metal dissolution reaction is weak [19,33,34,35]. Steel No. 9 with 0.0692% niobium has the largest arc reactance radius compared to other experimental steels in the initial corrosion stage. Consequently, the corrosion resistance of steel No. 9 is the best in the initial corrosion stage.

The Bode plots reflect the relationship between the impedance modulus, phase angle, and frequency. Figure 5b shows the characteristics of the relationship between the impedance modulus and frequency. The impedance modulus measured in the highest frequency region in the Lg Z—Lg ω Bode plots represents R_s_. The impedance modulus measured in the lowest frequency region represents the sum of R_s_ and R_t_. The information shown in Figure 5b is consistent with that shown in Figure 5a. The solution resistance R_s_ changes slightly; however, the sum of R_s_ and R_t_ gradually increases with increasing niobium content, except for steel No. 9. The (R_s_ + R_t_) of steel No. 9 is the largest. R_t_ is inversely proportional to the degree of electrochemical reaction [30,36]. Therefore, Figure 5b also shows that, in steel No. 8, 10, and 11, the charge transfer resistance of the corrosion reaction increases, and the degree of corrosion reaction decreases with increasing niobium content.

The phase angle—Lg ω curves of the four ULCB steels still have similar characteristics. A distinct peak exists in the low-frequency part and a small hump in the middle and high frequency parts. The small hump of steel No. 9 and 11 in the middle and high frequency areas are more evident than those of steel No. 8 and steel No. 10. The corrosion process appears as two-time constants. This implies that the electrochemical reaction of the four types of steels was affected by two state variables, the double electric layer and corrosion products. The equivalent circuit for the test steel is illustrated in Figure 5d. Here, R_s_ represents the solution resistance; C_c_ and R_c_ refer to the capacitance of the corrosion product film and its resistance, respectively; C_d_ is the capacitance of the double electrode layer; and R_t_ stands for the charge transfer resistance.

The charge transfer resistances of steel No. 8, 9, 10, and 11 calculated analytically after fitting are 523.3, 1148.0, 1090.4, and 1116 ohm·cm^2^, respectively. The fitting results are consistent with the curve characteristics presented in the Bode plots. R_t_ values increase with increasing niobium content. It is further proved that, except for steel No. 9, the charge transfer resistance of other steels increases, and the degree of corrosion tends to decrease with increasing niobium content. Steel No. 9 with 0.0692% Nb has the largest charge transfer resistance compared to other experimental steels in the initial corrosion stage. This conclusion is consistent with the conclusion drawn from the polarization curve.

### 3.5. Corrosion Rate Curves and Surface Corrosion Morphology of Four Experimental Steels

**Figure 6:**
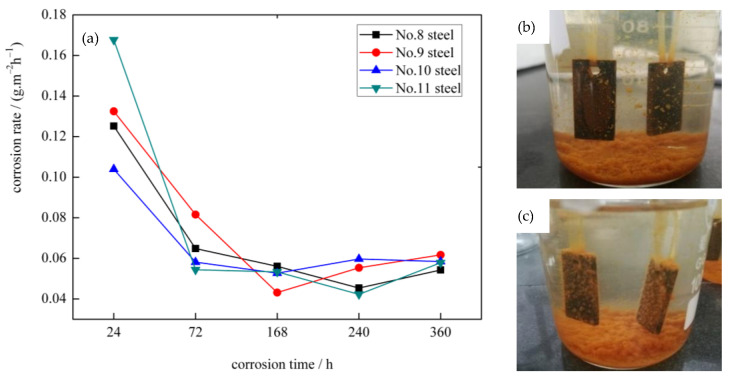
(**a**) The corrosion rates curves of four types bainite steels in 3.5 wt.% NaCl aqueous solution and corrosion phenomenon observed of (**b**) No. 10 steel and (**c**) No. 11 steel was soaked in 3.5 wt.% NaCl solution with 168 h.

The weight-loss method is the most basic and effective quantitative evaluation method in the study of corrosion resistance of materials. The corrosion rate curves of the four types of bainite steels in 3.5 wt.% NaCl solution based on gravimetric experiment results are demonstrated in Figure 6a. The corrosion phenomenon observed in steel No.10 and 11 was soaked in 3.5 wt.% NaCl solution for 168 h is shown in Figure 6b,c. As shown in Figure 6a, the corrosion rates of the four types of ULCB steels are approximately the same in general trend. Within 0–360 h of corrosion time, the corrosion rate of the four types of steels is relatively high during the initial immersion stage. With the increase in immersion time, the corrosion rate gradually decreases and becomes stable; then, the corrosion rate increases slightly. Although the overall corrosion rate trend is similar, the time nodes of the corrosion rate valley values of the four types of steels are different.

Within the immersion time range of this experiment, the corrosion rates of the four types of steels reached the maximum after soaking for 24 h. At this point, the corrosion rate of steel No.11 was the highest, and that of No.10 was the lowest. After soaking for 72 h, the corrosion rates of the four types of steels decreased significantly, among which the lowest one was steel No.11. The corrosion rates of steel No.9 and 10 reached the minimum after 168 h of immersion. After soaking for 240 h, the corrosion rates of steel No.8 and 11 reached the minimum. Meanwhile, the corrosion rates of steel No.9 and 10 increased slightly. After soaking for 360 h, the corrosion rates of steel No.8 and 11 increased slightly, and those of steel No.9 and 10 tended to be stable. The corrosion rates of the four types of steels showed slight difference when immersed for 360 h.

During the experiment, it was observed that the solution in the beaker turned slightly yellow after the sample was soaked for approximately 1 h. After 24 h of corrosion, corrosion precipitation clearly contained in the beaker. The solution had completely turned yellow. Distinct corrosion occurred on the surface of the sample. After 168 h of corrosion, the surface of the sample was full of corrosion products, and the bottom of the beaker was full of corrosion precipitate, as shown in Figure 6b,c. If the beaker shakes slightly, some corrosion products on the sample surface fall off and leak out the corrosion morphology of the sample surface.

**Figure 7:**
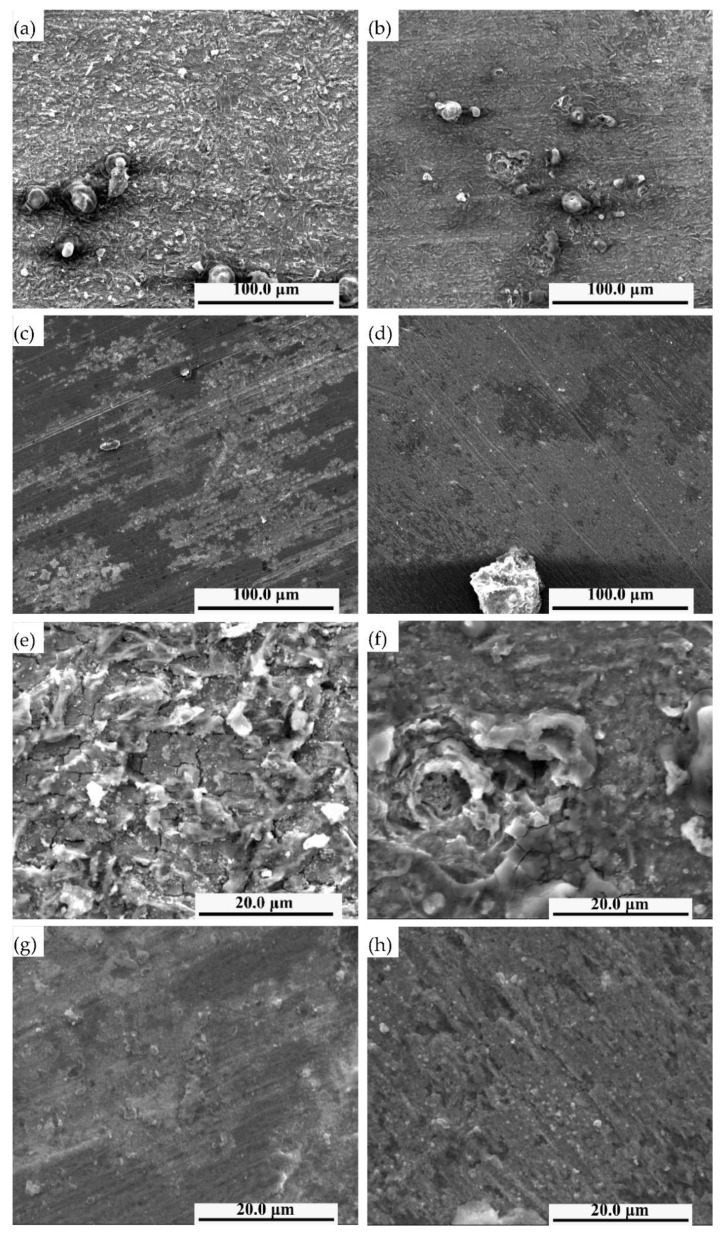
Surface corrosion morphology of No. 8 steel (**a**,**e**), No. 9 steel (**b**,**f**), No. 10 steel (**c**,**g**) and No. 11 steel (**d**,**h**) after 240 h corrosion.

The surface corrosion morphologies of the four types of steel samples after 240 h of corrosion are shown in Figure 7. It is observed that the surface corrosion products of steel No.10 and 11 with higher niobium content are much smoother and denser than those of steel No.8 and 9 with lower niobium content when the experimental steels undergo corrosion for 240 h, on the whole. In particular, the white corrosion products on the surface of steel No.8 are uniformly distributed as islands and are denser. There is more granular material, and the protrusion is more evident, as shown in Figure 7a. After further magnification, the white corrosion products of steel No.8 are in the form of debris, and there are evident cracks in the middle of the corrosion products with a large number of cracks and local corrosion pits, as shown in Figure 7e. In steel No.9, there are significantly fewer white corrosion products and lower convex height of granular material compared to steel No.8. A small number of short shallow cracks and local corrosion pits are present in the corrosion products, as shown in Figure 7b,f. As shown in Figure 7c,g, the surface of the corrosion products of steel No.10 is relatively smooth and dense, and cracks are almost invisible; the number and size of white corrosion products are small, and the protrusions are not evident. The corrosion product of steel No.11 is slightly loose than that of steel No.10. As shown in Figure 7d,h, there are no large white corrosion products on the surface of steel No.11; only a few small white spots exist, and no cracks can be seen in the corrosion products.

## 4. Discussion

### 4.1. Correlation between Microstructure, Mechanical Properties, Corrosion Behavior of ULCB Steel, and Nb Content

From the experimental results, it can be concluded that niobium content plays a vital role in the microstructure, mechanical properties, and corrosion properties of ULCB steel.

Nb microalloying can be beneficial for achieving finer grain structure. The equilibrium dissolution temperature of the carbonitride of niobium precipitation can be calculated by the following equation [37]:

(2) log [Nb][C+12N/14]=2.26−6 770/T

where [Nb], [C], and [N] are the elemental contents of niobium, carbon, and nitrogen (mass %), respectively, and T refers to the temperature (K).

The calculated equilibrium dissolution temperature of the carbonitride of niobium precipitation of the four types of steels is approximately 1076.3 °C, 1160.5 °C, 1186.6 °C, and 1220.8 °C, respectively. The calculated results exceed the initial rolling temperature of 1050 °C. Niobium is precipitated in steel in the form of carbonitrides. Carbonitride of niobium precipitation can control the austenite grain size during reheating, retard recrystallization during deformation, and provide precipitation strengthening during cooling. In a certain range of niobium content, with the increase in niobium content, the precipitates increase and the effect of strengthening and toughening is excellent. However, too much niobium content leads to the coarsening of precipitates, which is not conducive to the improvement of strengthening and toughening.

ULCB steel with higher Nb content has evident advantages in inhibiting the coarsening of the original austenite grains and maintaining the grain uniformity, which is beneficial to the subsequent phase transition. Moreover, Nb addition is also more conducive to the generation of bainite microstructure. As the niobium content increases, the microstructure of ULCB steel undergoes transition from granular bainite to Lath bainite. The content and size of the M/A component in the microstructure also changes with niobium content.

These multiplex effects decrease the grain size of the microstructure after transformation and improve the strength and toughness of the steel [25]. It also compensates for the loss of strength arising from the lower carbon content of the ULCB steel.

Moreover, by adding trace Nb to improve the strength and toughness of materials, the use of other elements can be reduced. The carbon equivalent decreases accordingly. A low carbon equivalent ensures better toughness and excellent weldability. The influence of Nb content on the steel weldability is on both sides [21,22], and the appropriate content of niobium is critical. The amount of niobium added needs to be moderate in range. It may refer to the American Petroleum Institute (API) Specification (Spec) 5 L standard [38] for the amount of niobium, vanadium, and titanium in micro-alloyed pipeline steel. The maximum Nb content in micro-alloyed pipeline steel can reach 0.15% in the absence of vanadium and titanium. However, it has also been suggested that the niobium content limit in offshore steel is extremely strict compared to pipeline steel [20].

Furthermore, Nb addition can reduce the carbon content of ULCB steel, change the chemical composition, and affect the microstructure, thus directly or indirectly affecting the corrosion performance.

When the steel is immersed in seawater, galvanic corrosion is formed between the ferrite (anode) and cementite (cathode) phases in the steel matrix. The reduction of carbon content can hinder the formation of cementite (Fe_3_C). Correspondingly, the associated galvanic corrosion associated with Fe_3_C reduces, resulting in a lower corrosion rate. In particular, for offshore platform steels, the addition of alloying elements such as Cu, Ni, and Cr can effectively increase the corrosion resistance [16,17,19]. Changes in chemical composition can change the transformation behavior of steel and the corresponding microstructure. Composition and microstructure play an extremely important role in determining the corrosion susceptibility of steel [11,26,39]. Metal composition can influence the first kinetically controlled corrosion phase [40].

Trace Nb affects the corrosion process of ULCB steel at different stages. At the initial stage of corrosion, a certain amount of Nb can improve the corrosion resistance of low-carbon bainite steel. This is because Nb can improve the uniformity of steel and increase the grain boundary with a small angle. However, the corrosion process of steel is essentially an electrochemical process; therefore, the reaction rate depends largely on the potential difference between the anode and cathode. Improving the uniformity of the steel structure is equivalent to reducing the potential difference between the anode and cathode of the corroded battery, thus reducing the corrosion rate. In contrast, low-carbon bainite steel has low interfacial energy and relatively low impurity concentration; therefore, there is no evident interfacial corrosion between the laths.

At the beginning of bare steel corrosion, except for steel No.9, the steel No.8, 10, and 11, exhibited the trend of increase in arc radius of the Nyquist plots increased with increasing niobium content. This indicates that the charge transfer resistance in the corrosion reaction increases and the current generated in the corrosion reaction decreases with increasing niobium content.

With the increase of corrosion time, ULCB steel with higher niobium content has a more denser rust layer, which can hinder the migration of ions and slow down the corrosion of the internal matrix, thus improving the corrosion resistance.

The reason for steel No.9 to have the largest capacitive reactance radius may be that the corrosion reaction is related not only to the chemical composition of the steel itself, but also to the structure, surface, and environmental media of the steel. When the surface state and environmental medium are similar, the main influencing factors are the composition and microstructure. Steel in NaCl solution has both macro- and micro-corrosion activities. The change in microstructure has a great influence on the micro-corrosion. In the initial corrosion stage without effective rust layer protection, micro-corrosion accounts for a large proportion. Theoretically, in ULCB steel, ultra-low carbon content can decrease corrosion rates, as mentioned above. However, specific to different micro-alloyed steel, the change of trace niobium content in their composition is not only the change of composition itself, but also the corresponding change of steel microstructure. The most evident changes are the microstructure type, grain size and shape, precipitation, size and distribution of the carbo-nitrate, as well as the shape, size, and distribution of M/A components. These changes undoubtedly affect the micro-corrosion. Therefore, the effect of the niobium content change is not linear, but complex.

In summary, there is no doubt that the change in niobium content can significantly affect the mechanical properties and corrosion resistance at the same time when other technological factors remain unchanged during the steel production.

### 4.2. Relationship between Corrosion Behavior of the Four Types ULCB Steels with Different Nb Content and the Evolution of the Corrosion Product Layer

The corrosion behavior of the four types of steels is closely related to the evolution of the rust layer. As mentioned previously, the corrosion rate of the four types of steels is relatively high in the initial immersion stage and then gradually decreases and becomes stable. The rust layer generated on the metal surface and the thickness, density of the rust layer, and crack in layer can be responsible for interpreting this trend. The effect of corrosion products on the macroscopic corrosion process can be divided into two stages: at the early corrosion stage, the thin and loose corrosion products on the steel surface cannot protect the matrix effectively, corrosion rate increases with corrosion time. At the later corrosion stage, the formation of a compact, continuous, and uniform corrosion product layer can protect the steel substrate from direct contact with the corrosion solution.

When the experimental steel was immersed in the NaCl solution, a galvanic corrosion cell was formed. The metal dissolution reaction occurred in the anode region, and a low potential region was formed on the metal surface; oxygen reduction and reduction reaction of corrosion products occurred in the cathode region, and a high potential region was formed. At the early stage of corrosion, there was no protective layer on the surface, and the exposed steel plate contacted the corrosion liquid rich in oxygen and Cl^-^ directly. Consequently, the dissolution of iron is fast, and the corrosion rate is exceedingly high. Then, a layer of corrosion products starts to form on the surface of the steel as the corrosion reaction progresses. At an early stage, the corrosion product layer is quite thin and loose. Dissolved oxygen in seawater passes through the corrosion product layer to reach the corrosive interface readily. At this point, the corrosion process is predominantly determined by the concentration of dissolved oxygen transferred from the solution medium to the metal surface. As more dissolved oxygen enters the metal interface to participate in the cathode reduction, the corrosion rate is elevated. The corrosion driving force is the potential difference between the anode and cathode on the surface at this stage. Therefore, the corrosion rate of the experimental steel in the initial immersion stage is relatively high.

When steel No.9 and 10 were soaked for 168 h, steel No.8 and 11 soaked for 240 h, the corrosion rate increased slightly. This phenomenon can be attributed to two reasons. One reason is that the rust layer formed at this time is too thin to prevent the entry of dissolved oxygen; inversely, its porous and loose structure provides a channel for dissolved oxygen to contact the metal matrix, so the corrosion rate increases. The other reason is the cracks generated in the corrosion products layer. At the early corrosion stage, the generated iron oxide generally maintains a certain coherent relationship with the steel matrix, reducing the surface energy and increasing the elastic strain energy of the interface. However, the corrosion product layer itself is brittle and difficult to deform. Therefore, cracks easily occur in the corrosion-producing layer, which provides a convenient channel for dissolved oxygen and Cl^-^ to further erode the matrix. Consequently, the thin, porous, and cracked rust layer has no protective effect, which is the main reason for an increase in the corrosion rate at this stage.

Combined with the surface corrosion morphology analysis, the sample steel No.8 showed a rougher surface and more white corrosion products than other steels after 240 h of corrosion in the simulated seawater, indicating that steel No.8 underwent serious corrosion in the corrosive liquid. From the corrosion morphology, the degree of corrosion of steel with higher niobium content is lower after 240 h of corrosion.

From the corrosion rate curve, steel No.11 with the highest niobium content has a faster corrosion rate at the initial corrosion stage. The main reason may be that steel No.11 has a small grain size and higher interfacial energy; thus, it shows a higher corrosion rate in the early stage. However, it can form a denser film that can inhibit further increases in the corrosion rate in a short time. The existing data indicate that the initial corrosion characteristics of steel are correlated with long-term corrosion behavior. If the bare steel corrodes uniformly and slowly, it may be beneficial to the tightness and stabilization of the corrosion product layer; that is, the corrosion behavior of bare steel is still transmissible after the formation of the rust layer. This may explain the reason for the highest initial corrosion rate of steel No.11 that reduces later.

For steel No.8 and 9, the corrosion rates of steel No.8 and 9 decreased rapidly after the formation of the corrosion product protective film at the early corrosion stage. The corrosion rates of steel No.8 and 9 minimized after 240 h and after 168 h, respectively, and there was a slight increase in the later stage. This can be explained by the sparse and scattered distribution of corrosion products and the existence of pores and cracks between the corrosion products, as shown in Figure 7.

The corrosion rates of the four types of bainitic steels gradually become stable after 360 h immersion, which is due to the formation of a protective corrosion product layer. With the increase in soaking time, the rust layer gradually covered the entire sample surface and the thickness gradually increased. The rust layer prevented Cl^-^ from contacting the metal matrix by setting obstacles in the transmission of dissolved oxygen to the matrix. Oxygen depolarization corrosion has been slightly inhibited. At the same time, the existence of a rust layer also prevents the iron ion from diffusing outward from the corrosion boundary. The increasing thickness of the surface rust slows down the diffusion process, eventually leading to a gradual decrease in the corrosion rate and a gradual reduction in the potential difference between the anode and cathode. The corrosion reaction rate of the corrosive liquid and bainite structure reached a dynamic balance with the inhibition rate of the corrosion layer, which inhibited further deepening of the corrosion phenomenon. At this time, a tight structure was beneficial for isolating the steel matrix from the corrosion solution.

The novelty of this work is the effect of niobium content on the microstructure, mechanical properties, and corrosion behavior of ULCB steel. It is well understood that both the composition and microstructure have a strong influence on the mechanical properties and corrosion behavior of steel. The present work studied the effect of niobium content on the microstructure, mechanical properties, and corrosion behavior of steel separately without the influence of the chemical composition of the steel and processing.

## 5. Conclusions

Four types of ULCB steels with different Nb contents were designed and produced. The correlation between the microstructure, mechanical properties, corrosion behavior of ULCB steel, and Nb content were investigated. The main conclusions drawn are as follows:

The microstructure of the four types of ULCB steels is mainly granular bainite with some martensite/austenite (M/A) components. The niobium content affects bainite morphology and the size, quantity, and distribution of M/A group elements.

All experimental steels in the as-rolled state have better plasticity and higher strength but lower impact toughness at −40 °C. The yield strength (R_el_), tensile strength (R_m_), and elongation (δ) of the four types of steels are all above 500 MPa, 650 MPa, and 20%, respectively. However, the impact toughness at −40 °C (A_KV_) of the four types of experimental steels in the as-rolled state is lower than 10 J. Steel No.9 with 0.0692% niobium has a better comprehensive property of elongation and strength.

Based on the weight-loss experiment results, the corrosion rate of the four types of steels is relatively high during the early immersion stage. With the increase in corrosion time, the corrosion rate gradually decreases and becomes stable; then, the corrosion rate increases slightly.

Steel No.9 with 0.0692% niobium has the largest charge transfer resistance compared to other experimental steels at the initial corrosion stage according to the polarization curve and impedance spectrum of bare steel. The surface of steel with higher niobium content has smoother and denser corrosion products compared to the steel with lower niobium content after 240 h of corrosion. The degree of corrosion decreases gradually with the increase in niobium content at the later stage of corrosion can be drawn by combining the corrosion rate curve and the morphology of the corrosion products.

## Figures and Tables

**Table 1:** The chemical compositions of the four types of experimental steels with different niobium contents (wt.%).

Steel	C	Si	Mn	P	S	Nb	Ni	Cr	Mo	Ti	Cu
No.8	0.047	0.20	1.59	0.009	0.006	0.035	0.615	0.413	0.249	0.015	0.29
No.9	0.05	0.21	1.55	0.008	0.006	0.069	0.596	0.410	0.252	0.014	0.30
No.10	0.05	0.19	1.54	0.008	0.007	0.084	0.600	0.430	0.240	0.011	0.343
No.11	0.05	0.197	1.56	0.009	0.007	0.107	0.582	0.450	0.241	0.013	0.336

**Table 2:** Fitting results of polarization curves.

Steel No.	No.8	No.9	No.10	No.11
I_0_ (Amp/cm^2^)	2.32 × 10^−5^	1.57 × 10^−5^	1.71 × 10^−5^	2.28 × 10^−5^
E_0_ (V)	−0.4578	−0.6084	−0.645	−0.665

## Data Availability

The data presented in this study are available on request from the corresponding author.
